# Development and Validation of a Lighting Facility for the Objective Assessment of the Visual Performance of Presbyopic Patients in a Series of Activities of Daily Living

**DOI:** 10.7759/cureus.24548

**Published:** 2022-04-28

**Authors:** Georgios Labiris, Eirini-Kanella Panagiotopoulou, Panagiota Ntonti

**Affiliations:** 1 Department of Ophthalmology, University Hospital of Alexandroupolis, Alexandroupolis, GRC

**Keywords:** near vision, intermediate vision, presbyopia, light temperature, light intensity, activities of daily living

## Abstract

Introduction

The primary objective of this study is to develop and validate an experimental lighting facility that allows the evaluation of near and intermediate vision in different user-defined illuminance levels.

Methods

This is a prospective, randomized, controlled study. Normophakic patients populated three validation groups (VGs) according to their binocular uncorrected near visual acuity (UNVA): a) VG1, 0.0-0.1 logMAR; b) VG2, 0.4 logMAR; and c) VG3, 0.7 logMAR. All participants addressed 10 near and intermediate activities of daily life (ADLs) in the three following lighting settings: 1) 25 foot candles (fc)/3000 kelvins (K), 2) 50 fc/4000 K, and 3) 75 fc/6000 K.

Results

Thirty patients in each group performed all ADLs in the three lighting settings. VG1 demonstrated the best ADL scores in all ADLs and lighting settings, followed by the VG2. VG3 presented the worst scores. ADLs using printed material showed significant differences among the three lighting settings for all study groups, while ADLs using screens or needing manual dexterity demonstrated no significant differences except for the Screwdriver Test (ST) in VG1. All ADL scores demonstrated a high correlation with UNVA in all lighting settings (p < 0.001).

Conclusion

This is the first study that validates a lighting facility for comparative studies in patients with different near vision capacities performing a series of ADLs.

## Introduction

Presbyopia is an age-related condition that results in reduced ability to perform certain activities of daily living (ADLs) at near and intermediate distances [1,2]. There are many theories regarding the causes of presbyopia, with the dysfunction of the accommodation system being the most prevalent [3]. Symptoms usually manifest in the late 40s, with gradually increasing difficulty in certain tasks accompanied by fatigue and headaches [4].

The prevalence of presbyopia is predicted to reach 1.8 billion by 2050 [5]. Contemporary correction methods include both surgical and conventional options. Spectacles and contact lenses are among the popular conventional treatment options; however, a significant proportion of presbyopic patients experience a significant reduction in their quality of life (spectacles) or ocular discomfort (contact lenses) [6,7]. Surgical options for presbyopia include laser and non-laser-assisted techniques that target the cornea and premium intraocular lens implantation in an attempt to restore the pre-presbyopic functionality of the ocular system [8-10].

Literature suggests that visual capacity depends heavily on environmental lighting [11,12]. Therefore, the American Illuminating Engineering Society (IES) has introduced directives regarding the recommended artificial lighting conditions in certain ADLs in an attempt to maximize the overall performance of the patient in the corresponding task [13]. However, IES lighting directives have not been validated in experimental settings with presbyopic patients. In fact, there is growing evidence that certain IES lighting directives result in suboptimal visual capacity and other IES directives to energy-wasting and pollution [14].

Within this context, the primary objective of this study is to develop and validate an experimental facility that will allow the evaluation of near and intermediate vision in different illuminance scenarios that are commonly encountered in public, home, and work settings.

## Materials and methods

Setting

This is a prospective, randomized, controlled study. The study protocol adhered to the Declaration of Helsinki, while written informed consent was provided by all participants. The scientific board of the Democritus University of Thrace approved the study protocol. The study was conducted at the Department of Ophthalmology in the University Hospital of Alexandroupolis, Greece, between July 2020 and June 2021. The official registration number of the study is NCT05005624.

The present study consisted of the following two main parts: a) the construction of the experimental lighting facility that enables the assessment of visual performance in different user-defined lighting scenarios and b) the validation of the facility for a series of simulated ADLs that require near and intermediate vision capacity.

Participants

Normophakic patients who visited our outpatient service were enrolled on a consecutive-if-eligible basis and populated the following three distinct validation groups (VGs) according to their binocular uncorrected near visual acuity (UNVA): a) validation group 1 (VG1) (30 participants) with binocular UNVA 0.0-0.1 logMAR, b) validation group 2 (VG2) (30 participants) with binocular UNVA 0.4 logMAR, and c) validation group 3 (VG3) (30 participants) with binocular UNVA 0.7 logMAR. The inclusion criteria included the following: a) adults older than 40 years old, b) clear crystalline lens, and c) fluency in written and verbal Greek language. The exclusion criteria included astigmatism >1.00 diopters, glaucoma, intraocular power-lowering medications, former incisional eye surgery, corneal or fundus disease, diabetes mellitus, autoimmune diseases, neurological, psychiatric or mental diseases, and inability to understand the objectives of the study.

Construction of an experimental lighting facility

An experimental facility at the University Hospital of Alexandroupolis was constructed for the sake of this study. In a hospital room with a dimension of 6.87 × 2.9 × 3 m (depth × width × height) and flat white surface walls (reflectance: 70%), an advanced light diffusion system was installed, which consisted of four linear LED luminaires producing low glare (unified glare rating (UGR) < 19). Light intensity (dimming) and light temperature (white tuning) were adjusted using the Casambi wireless control application (Casambi Technologies Oy Inc., Espoo, Finland), which uses integrated Bluetooth mesh technology and secures maximal uniformity at different user-defined lighting settings.

**Figure 1:**
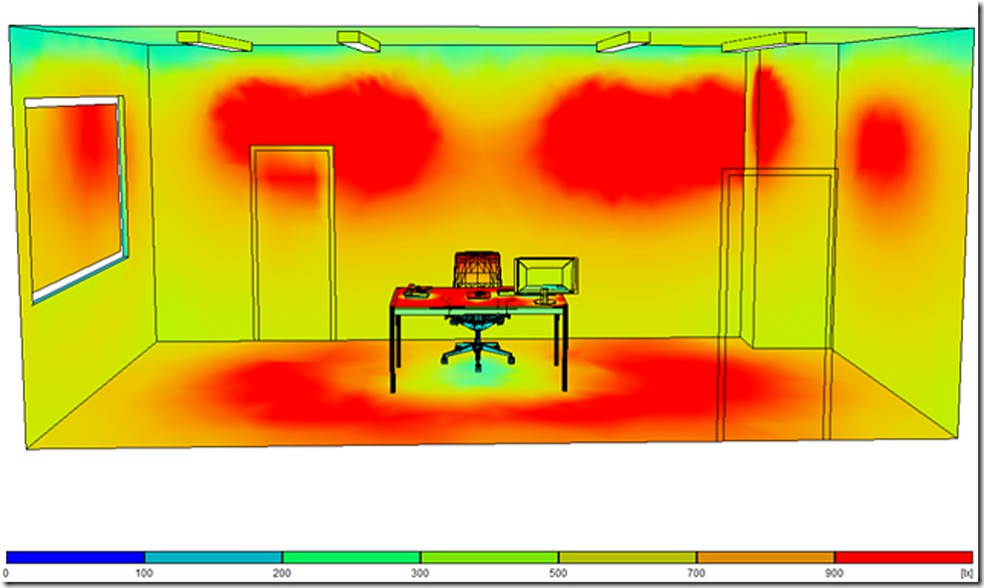
A three-dimensional (3D) illustration of the examination room with the four luminaires installed on the ceiling at their actual positions. The light intensity on the surfaces inside the room is overlaid and encoded in color according to the chromatic scale shown at the bottom (units: lx) using the RELUX software.

**Figure 2:**
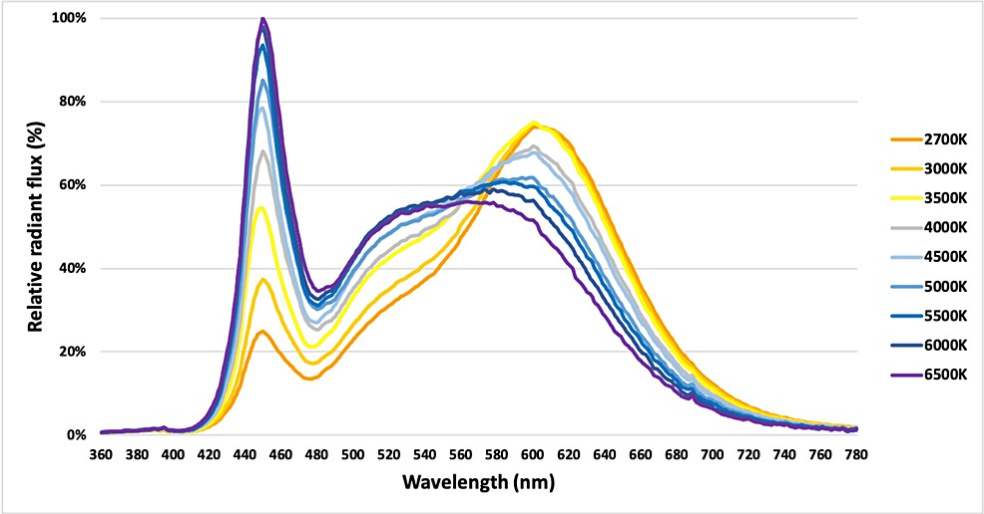
Relative radiant flux distribution of the luminaires in the visible spectrum for a range of light temperatures.

The four LED luminaires were mounted on the ceiling. The exact luminaire positioning and the amount of the provided luminous flux were defined using the RELUX light simulation tool (﻿version 2021.1.1.0) (Relux Informatik AG, Münchenstein, Switzerland) prior to the installation (Figure 1) [15]. The confirmation of illuminance and on-site adjustments were confirmed with the Extech Lux Meter EA30 (Extech Instruments Corporation, Nashua, NH, United States). As regards photometric properties derived from ﻿lighting laboratory photometric measurements, the correlated color temperature (CCT) of the luminaires ranged between 2700 kelvins (K) and 6500 K, emitting a maximum luminous flux of 10,626 lm and 11,508 lm, respectively. The exact radiant flux P(λ) emitted from the selected light sources was measured using a Konica Minolta CL-500A spectrum meter for the wavelength range of 380-780 nm using a step of 1 nm (Figure 2). The power of each luminaire was 106 W, resulting in luminous efficacy of 100.2 lm/W and 108.6 lm/W for 2700 K and 6500 K, respectively. The color rendering index (CRI) was 84. The wireless dimming control system enabled dimming from 0% to 100% and vice versa.

Using warm light (2700 K) and the maximum light output of the luminaires (dimming level: 100%), the calculated average illuminance on the overall working plane (task area level: 0.8 m) was 1100 ± 35 lx, and 700 ± 29 lx at the vertical plane (walls) with a maintenance factor of 0.8. Additionally, using cool light (6500 K) and the maximum light output of the luminaires, the calculated average illuminance in the overall working plane was 1250 ± 41 lx and 790 ± 32 lx at the vertical plane with a maintenance factor of 0.8.

Activities of daily living framework

A former report from our research group introduced an ADL framework for the fundamental assessment of near and intermediate vision intensive tasks in specific lighting conditions (task lighting: 3000 K - 80 foot candles (fc) in workspace; ambient lighting: 3000 K - 70 fc) (Table 1) [16]. However, in real-life settings, people have to address ADLs in a variety of lighting conditions. It has been found that light intensity and light temperature do influence near and intermediate vision capacity. Within this context, ADLs that simulate daily tasks should be validated in different lighting settings that people commonly encounter in their routine daily life in the following way. Each ADL requires a certain capacity for near or intermediate vision. The vision capacity for each ADL can be measured by the time required to complete a task (task duration) and the number of errors made during the task. Therefore, two general scales that standardize the participants’ score for any given ADL using the task duration parameter and/or the number of error parameters were introduced.

**Table 1:** Detailed description of activities of daily living (ADL). Task duration (in seconds) ^a^Cellular screen specifications: screen resolution 720 × 1280 pixels; pixel density ~294 ppi; screen brightness 100% ^b^Computer screen specifications: 13.3-inch screen; screen resolution 3840 × 2160 pixels; pixel density 331.3 ppi; Times New Roman font size 12; zoom 150%; brightness 100%; contrast 50%

ADL	Description	Measured parameter
Phone Book Search (PBS)	Patient is required to find and read a specific entry in a regular phone book catalog in the three different lighting settings. A different entry is asked to be found in each combination.	Task duration
Supermarket Receipt (SupRe)	Patient is required to find and read three products from a typical supermarket receipt (monospaced Sans Serif font) in the three different lighting settings. Different products are asked to be found in each combination.	Task duration + (number of errors × 2)
Book Reading (BR)	Patient is required to read an excerpt of a predetermined length in a novel in the three different lighting settings. A different excerpt of the same length is asked to be read in each combination.	Task duration + (number of errors × 2)
Drops bottle Reading (DR)	Patient is required to correctly read the expiration date printed on three typical eye dropper bottles in the three different lighting settings. Different eye dropper bottles are used in each combination.	Task duration + (number of errors × 2)
Cellular Message (CM)	Patient is required to read a Short Message Service (SMS) on a 5-inch cellular phone^a^ in the three different lighting settings. A different SMS is asked to be read in each combination.	Task duration + (number of errors × 2)
Cellular Entry Search (CES)	Patient is required to find and read one specific entry on a 5-inch cellular phone^a^ in the three different lighting settings. Different entries are asked to be found in each combination.	Task duration
Reading Computer Screen (RCS)	Patient is required to correctly read text of a predetermined length from a computer screen^b^ in the three different lighting settings. Different texts of the same length are asked to be read in each combination.	Task duration + (number of errors × 2)
Subtitles Reading (SubRe)	Patient is required to correctly read movie subtitles from a one-minute movie clip on a computer screen^b^ in the three different lighting settings. Different movie clips are shown in each combination.	Number of errors
Open Door Test (ODT)	Patient is required to find a specific key from a keychain that holds 10 keys and insert it in the corresponding door keyhole in the three different lighting settings. Different keychains are given in each combination.	Task duration
Screwdriver Test (ST)	Patient is required to select one among three screwdrivers and insert it in the appropriate screw among a variety of screw types in the three different lighting settings. Different screwdrivers are given to be selected in each combination.	Task duration

The first scale uses the task duration or a combination of task duration with the number of errors, and the second scale is applied to the measured number of errors.

The two scales are fundamentally different since task duration is a continuous variable in range (0, ∞); thus, assessing task duration presents the difficulty of undefined absolute best and absolute worst performance. On the other hand, the number of errors is an integer variable in range $\left [ 0, n_{max} \right ]$, with well-defined lower and upper limits, equal to 0 and to *n_max_*, respectively (*n_max_*: the total number of words to be read for each ADL).

Definition of a Standard Timescale for Assessing ADL Task Duration

Let a timescale be defined, such that every n_0_ steps the corresponding time is multiplied by a constant factor *λ*. If *t_1_,* *t_2_* are two times that should be mapped in the defined scale as *n_0_* steps apart, with *t_1_* > *t_2_*, then *λ* = *t_2_* / *t_1_*. The relationship between the required time *t* and scale *s* is $t=t_{1}a^{s}$ (1), where *t_1_* is a time parameter to be determined and $\alpha =\lambda ^{\frac{1}{n{0}}}$.

Using Equation (1), the score *s* can be calculated given the time *t *as follows: $s=\frac{1}{lna}\cdot ln\frac{t}{t_{1}}$ (2).

Equivalently, when the score increases by one step, time decreases by a factor of 1/*a*. By convention, negative scores can be assigned to a 0 score.

Let us present an example to clarify the above and highlight its main properties. Let us assume an ADL task for which we wish to define a scale that maps time *t_1_* = 15 seconds and time *t_2_* = 3 seconds, with a difference of 10 steps. The values of *λ* and a can be easily confirmed: $\lambda =\frac{3}{15}=\frac{1}{5}, \alpha =\left ( \frac{1}{5} \right )^{\frac{1}{10}}=0.8513$ (3).

By substituting Equation (3) in Equation (2), the definition of the specific scale is obtained: $s=\frac{1}{ln0.8513}\cdot ln\frac{t}{t_{1}}$ (4).

**Figure 3:**
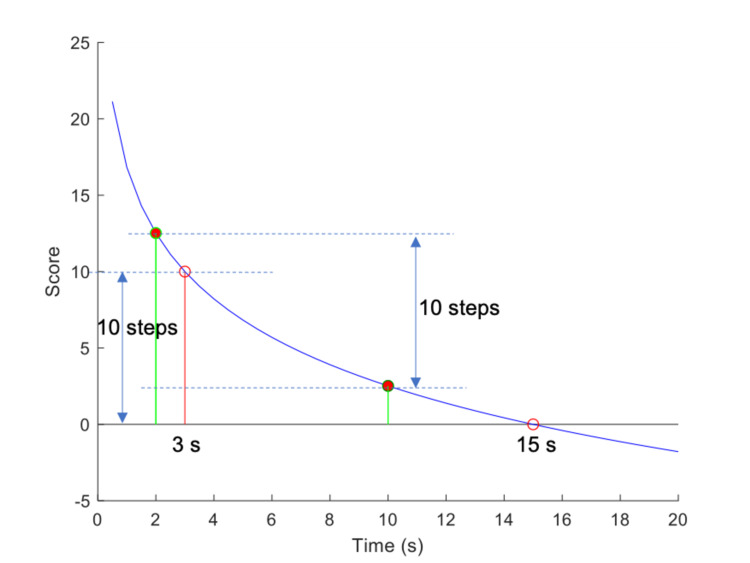
Graphical representation of the scale. Defined by setting λ = 1/5 and n_0_ = 10 (steps).

Figure 3 shows the score (in steps) against the time (in seconds) for the scale in this example. It can be observed that times *t_1_* = 10 seconds and *t_2_* = 2 seconds correspond to scale equal to 0 steps and 10 steps, respectively, which satisfies the definition of the scale. It is easy to observe that the ratio of any two times *t_a_*, *t_b_* with scores that differ by 10 steps is equal to 5. This property is also demonstrated in Figure 3, for *t_a_* = 15 seconds, *t_b_* = 3 seconds, whose scores *t_a_* = 2.5193 and *t_b_* = 12.5193 also differ by 10 steps.

For each ADL assessed by duration in this work, a scale was defined according to Equation (2), calculating parameters *t_1_* and *t_2_* as the average of the worst 5% and the best 5% time values required by all participants, respectively, whereas *n_0_* was set equal to 100.

Standard Exponential Scale for Assessing the Number of Errors in ADL Tasks

Let *n_1_* denote the average number of errors of the worst 5% of performing participants. The proposed error scale should map 0 errors to the scale’s best value (score equal to 100) and *n_max_* errors to a score close to 0. In practice, it has been observed that most of the participants achieve a number of errors much lower than *n_max_*. For instance, in SubRe ADL, it was found that *n_max_* = 135 and *n_1_* = 40. Consequently, a linear scale that maps the number of errors to the predefined range $\left [0, 100 \right]$ would not differentiate sufficiently between the majority of the participants, which would be squeezed at the high end of the scale. To solve this issue, we propose an exponential scale *s_n_* that will discriminate the participants’ number of errors *n* more strongly at the high end of the scale. More formally, the exponential scale for assessing the number of errors is defined as follows: $s_{n}=100e^{-bn}$ (5).

The parameter *b* is calculated by requiring *n_1_* to be mapped at score *s_n_* = 5 (selected experimentally): $b=\frac{-ln0.05}{n_{1}}$ (6).

**Figure 4:**
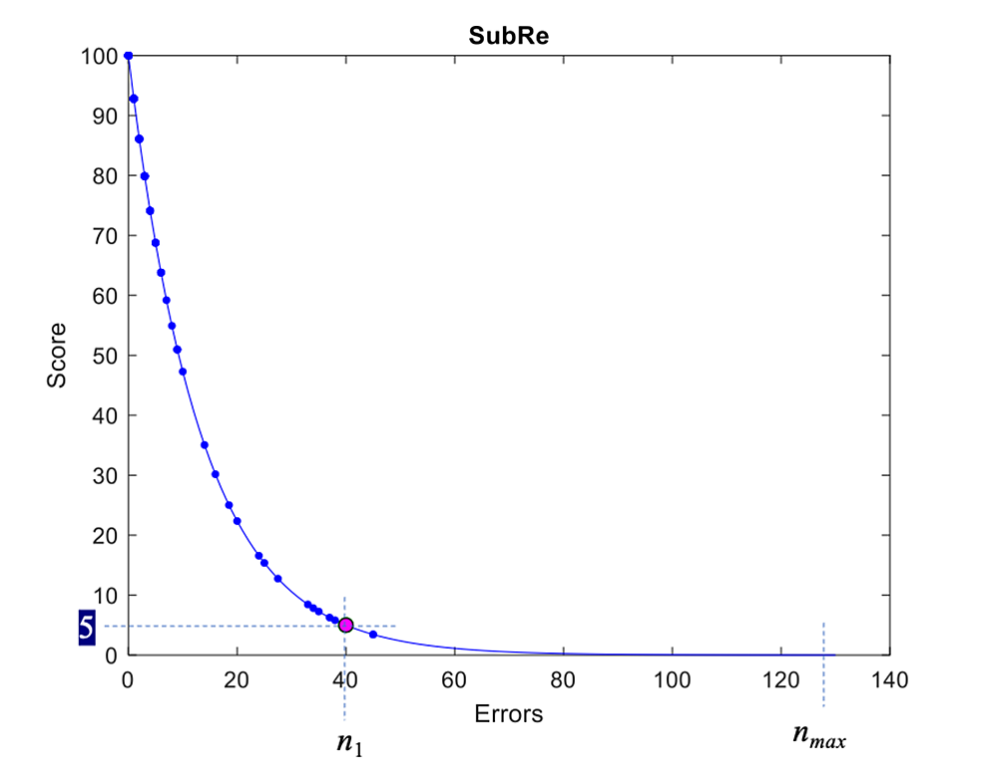
Graphical representation. Proposed s_n_ scale, applied to the SubRe ADL task.

The application of the proposed error scale to the number of errors during the SubRe ADL is shown in Figure 4, where the score of the *s_n_* scale is plotted against the number of errors according to Equation (5). The data points are shown as dots, whereas *s_1_* (errors) are mapped to score = 5, shown as a red circle. It can be observed that the best score on the scale is defined as 100 (contrary to the unbounded score of the timescale in Equation (2), where the score may become higher than 100 as the time-to-complete the task approaches 0). In theory, the minimum score will not become equal to 0; however, it can be confirmed that it will approach 0. More specifically, assuming that *n_max_* = *a* •*n_1_*, then the score for *n_max_* errors is equal to $s_{n}=100e^{-bn_{max}}=100e^{aln0.05}$ (7).

For instance, for *a* = 3, the score of *n_max_* becomes equal to 0.000125, which may be safely considered as 0 in the $\left [ 0, 100 \right ]$ scale.

If a participant was not able to complete a task, then the measured parameter (duration or number of errors) was set to a very high value, which leads the score to become equal to 0.

Data collection

All participants addressed the 10 ADLs in the following lighting settings: Lighting Setting 1, 25 fc/3000 K; Lighting Setting 2, 50 fc/4000 K; and Lighting Setting 3, 75 fc/6000 K). The order of the different lighting settings was randomly selected for each patient to reduce bias. A full examination in each lighting setting required an average of 30 minutes. The duration and/or the number of errors were measured by an independent researcher with no direct involvement in the study. The ADL score was calculated for all tasks based on the measured duration and/or number of errors, according to the appropriate scale defined in Equation (2) and Equation (5). In case a participant was unable to complete a task, the ADL score was 0.

The following clinical indices were evaluated: a) binocular uncorrected near visual acuity (UNVA), b) binocular uncorrected intermediate visual acuity (UIVA), both using the web-based Democritus Digital Acuity Reading Test (wDDART) [17,18], and c) spherical equivalent.

Statistical analysis

A power of 0.8 at the significance level of 0.05 for an effect size of 0.61-0.73 would be achieved using 24-30 participants, according to an a priori power analysis applied for each ADL score. The normality of the measured data was evaluated using the Shapiro-Wilk test.

The construct validity of the ADL score was assessed for the three lighting settings using the Kruskal-Wallis test to confirm that all ADL tasks could efficiently discriminate three validation groups based on their UNVA. The comparison among the three lighting settings for each validation group for all ADL scores was performed using the Friedman test. ADL scores were correlated with binocular UNVA using Pearson correlation. Test-retest reliability was assessed in the aforementioned validation groups by calculating intraclass correlation coefficients (ICCs) (two-way mixed model with measures of consistency, single rating) for all ADLs at the three combinations of light conditions in two different visits with an average 15-day time window to prevent memory effect. P-values < 0.05 were defined as statistically significant. All statistical analyses were performed using the MedCalc version 9.6.2.0 (MedCalc Software, Mariakerke, Belgium).

## Results

Calculation of ADL scores

The defined scales in Equation (2) and Equation (5) have to be parameterized for each ADL separately before they can be applied to the experimental data. More specifically, the timescale requires the value of time parameters *t_1_* and *t_2_* as the average of the worst 5% and the best 5% duration time values, respectively, required by all participants in the validation study, irrespectively of the vision group, light intensity, and light temperature. Consequently, the defined scale will cover an extended range of values. The parameter *n_0_* was set equal to 100.

The error scale was parameterized by calculating *n_1_* as the average of the worst 5% of the participants in the validation study (in terms of the number of errors) for each ADL separately.

The score of all participants for each ADL, both in the validation and the main study, were calculated using the scale definitions in Equation (2) and Equation (5).

Table 2 presents values of the coefficients *t_1_*, *t_2_*, *λ* and *a* of Equation (2) for each of the ADLs that are assessed using time duration. The only parameter *n_1_* of the error scale was calculated equal to 0.0749 for the SubRe ADL.

**Table 2:** Timescale coefficients for each ADL score. ADL: activities of daily living; BR: Book Reading; CES: Cellular Entry Search; CM: Cellular Message; DR: Drops bottle Reading; ODT: Open Door Test; PBS: Phone Book Search; RCS: Reading Computer Screen; ST: Screwdriver Test; SupRe: Supermarket Receipt

	t_2_	t_1_	λ	a
PBS	4.441	102.676	0.043253	0.969081
SupRe	8.472	76.416	0.110867	0.978246
BR	31.904	173.82	0.183546	0.983190
DR	5.531	40.970	0.135001	0.980174
CM	5.564	33.918	0.164043	0.982086
CES	4.146	37.678	0.110038	0.978172
RCS	26.884	122.486	0.219486	0.984950
ODT	4.658	26.628	0.174929	0.982717
ST	3.712	24.185	0.153484	0.981433

**Figure 5:**
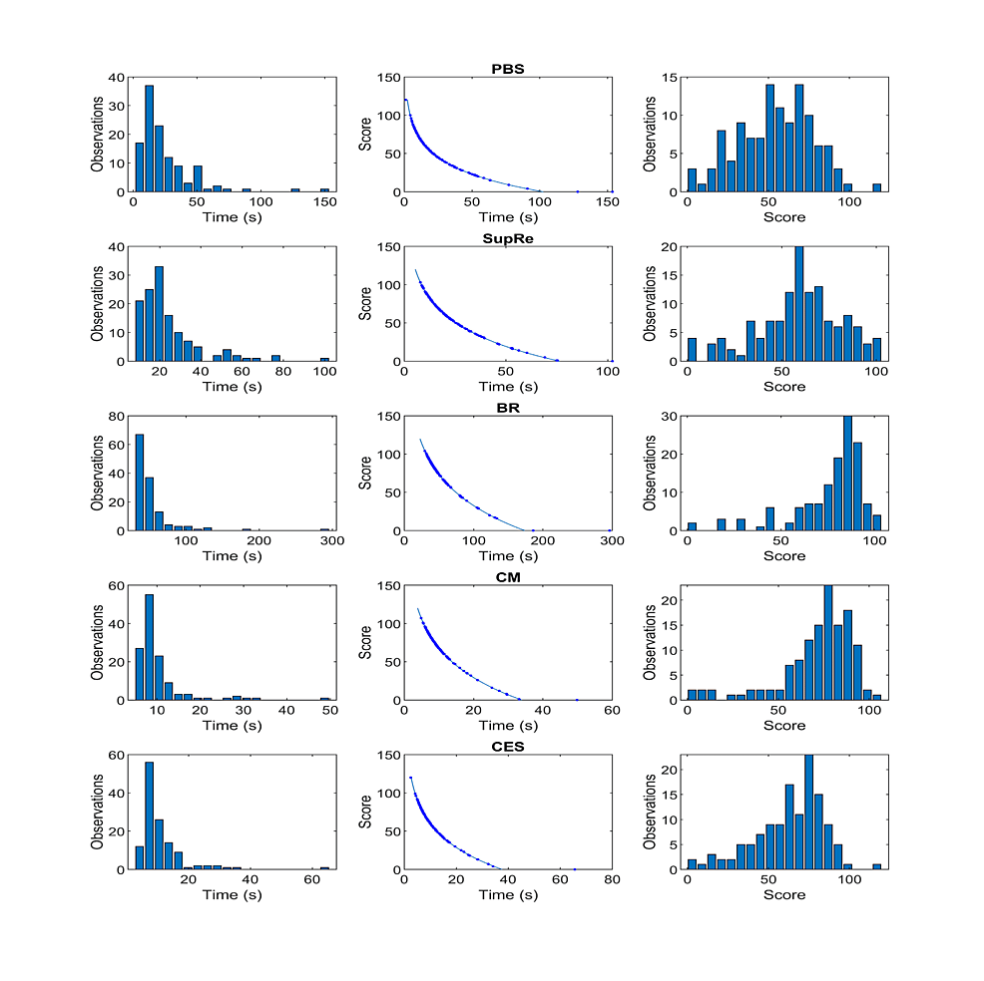
The application of the proposed scales to the first 5 ADLs. Left column: Histograms of time duration Middle column: Mapping from duration to the score of the defined time scale (data-points of validation study are overlaid). Right column: Histograms of the scale values of each ADL.

**Figure 6:**
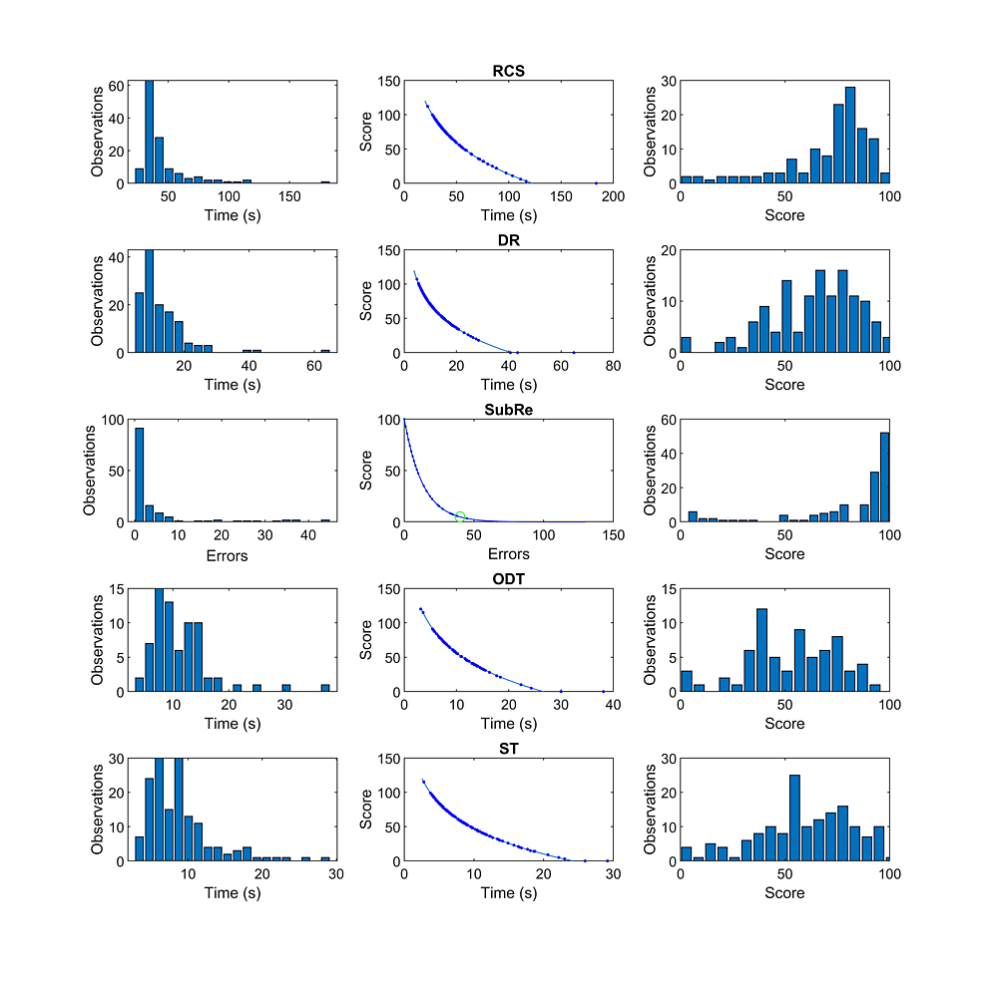
The application of the proposed scales to the rest 5 ADLs. Left column: Histograms of time duration (for the 4 ADLs) and histogram of the number of errors (for SubRe). Middle column: Mapping from duration or number of errors to the score of the defined time scale or error scale (data-points of validation study are overlaid). Right column: Histograms of the scale values of each ADL.

The histograms of time duration for the nine ADLs and the histogram of the number of errors for the SubRe ADL are shown in the left column of Figures 5 and 6 considering collectively all combinations of light intensity, light temperature, and validation group in the validation study. The mapping from the duration or number of errors to the score of the defined timescale or error scale, respectively, is shown in the middle column of Figures 5 and 6, with the data points of the validation study overlaid. The histograms of the scale values of each ADL are depicted in the right column. As was expected from the definition of the proposed timescale, very short durations are mapped to scores above 100, whereas very long durations correspond to negative scores, which by convention are set to 0. On the other hand, the error scale that is applied to SubRe ADL has a maximum value of 100 that corresponds to 0 errors, and it approaches 0 score asymptotically, as the number of errors increases. The score of the average of the worse performing 5% of participants in this task is shown as a green circle.

Validation study

The demographics of the three validation groups are presented in Table 3. A total of 90 people (n = 30 patients in each group; 46 men (51.11%) and 44 women (48.89%)) participated in the study, with an average age of 60.8 ± 8.63 years and spherical equivalent of -0.03 ± 0.86 D that populated the three groups. Significant differences were detected in UIVA (p < 0.001) and UNVA (p < 0.001), while nonsignificant differences were detected in age (p = 0.712) and spherical equivalent (p = 0.091).

**Table 3:** Demographics of the three validation groups. *p < 0.001 D: diopters; NA: not applicable; UIVA: uncorrected intermediate visual acuity; UNVA: uncorrected near visual acuity; VG: validation group

Parameters	VG1	VG2	VG3	P-value
N	30	30	30	NA
Gender (male/female)	17/13	15/15	14/16	0.733
Age (years)	57.33 ± 9.08	58.57 ± 5.71	60.71 ± 8.98	0.712
Spherical equivalent (D)	-0.69 ± 0.83	0.13 ± 0.60	0.46 ± 0.81	0.091
UIVA (logMAR)	-0.08 ± 0.06	0.10 ± 0.19	0.58 ± 0.10	<0.001*
UNVA (logMAR)	0.05 ± 0.07	0.43 ± 0.12	0.73 ± 0.08	<0.001*

The difference in the achieved scores by VG1, VG2, and VG3 is statistically significant according to the Kruskal-Wallis test for all ADLs under the same lighting settings. Thus, the ADL framework demonstrated sufficient construct validity since all ADL tasks could efficiently discriminate validation groups for all lighting settings (Table 4).

The ADL scores among the three lighting settings were also compared for each one of the three validation groups (Table 4). It can be verified that the median score of ADLs based on printed material (PBS, SupRe, BR, and DR) increased monotonically as the light intensity and light temperature increased for the VG1 and VG2. The Friedman test confirmed the statistical significance of the difference in ADL scores under the three different lighting settings. On the other hand, the achieved score for the ADLs using digital screens (CM, CES, RCS, and SubRe) and the ADLs that required manual dexterity (ODT and ST) did not show any dependence on lighting settings, except for ST in VG1. The low visual acuity of VG3 patients did not allow the majority of them to complete the ADLs; thus, a median score equal to 0 was calculated for all ADLs, except for ODT and ST. In these two tasks, the lighting settings appeared to have no statistically significant effect on ADL score, as it was indicated by the Friedman test.

**Table 4:** Comparison of ADL scores for the three light intensities among the three validation groups (VG1, VG2, and VG3). * p < 0.001, ^#^ p < 0.01, ^♭^p < 0.05 Lighting Setting 1: 25 fc/3000 K; Lighting Setting 2: 50 fc/4000 K; Lighting Setting 3: 75 fc/6000 K ADLs: activities of daily living, BR: Book Reading; CES: Cellular Entry Search; CM: Cellular Message; DR: Drops bottle Reading; N/A: not applicable; ODT: Open Door Test; PBS: Phone Book Search; RCS: Reading Computer Screen; ST: Screwdriver Test; SubRe: Subtitles Reading; SupRe: Supermarket Receipt; VG: validation group

ADLs	Lighting settings	VG1	VG2	VG3	P-value (Kruskal-Wallis test)
		Median (interquartile range)			
PBS	1	50 (33.13, 70.01)	0 (0, 0)	0 (0, 0)	<0.001*
	2	54 (38.33, 69.05)	0 (0, 16)	0 (0, 0)	<0.001*
	3	55 (39.34, 72.56)	0 (0, 33.40)	0 (0, 1)	<0.001*
P-value (Friedman test)		0.002^#^	0.046^♭^	N/A	
SupRe	1	60.43 (50.86, 67.88)	35.74 (7.87, 41.94)	0 (0, 0)	<0.001*
	2	63.04 (51.83, 72.78)	38 (25, 47.50)	0 (0, 1.50)	<0.001*
	3	65.66 (49.42, 77.15)	50.23 (41.44, 55.63)	0 (0, 6)	<0.001*
P-value (Friedman test)		0.015^♭^	<0.001*	N/A	
BR	1	81.31 (75.79, 86.27)	46.65 (7.76, 63.89)	0 (0, 0)	<0.001*
	2	84.16 (72.56, 89.05)	64.50 (27, 71.50)	0 (0, 0)	<0.001*
	3	85 (74.45, 89.21)	76.03 (44.48, 84.40)	0 (0, 2)	<0.001*
P-value (Friedman test)		0.002^#^	<0.001*	N/A	
DR	1	66.03 (48.94, 77.24)	30.42 (20.16, 41.16)	0 (0, 0)	<0.001*
	2	68.61 (55, 79.95)	32 (24.50, 47.76)	0 (0, 0)	<0.001*
	3	69.24 (60.23, 81.04)	44.01 (28.86, 50.21)	0 (0, 1)	<0.001*
P-value (Friedman test)		0.024^♭^	0.043^♭^	N/A	
CM	1	77.15 (63.77, 84.57)	58.07 (33.11, 72.33)	0 (0, 0)	<0.001*
	2	78 (68.15, 85.36)	62.75 (47.50, 72.24)	0 (0, 2.50)	<0.001*
	3	77.44 (66.34, 86.63)	69.47 (63.52, 70.99)	0 (0, 8)	<0.001*
P-value (Friedman test)		0.167	0.176	N/A	
CES	1	64.73 (51.50, 77.50)	60.85 (40.25, 72.84)	0 (0, 0)	<0.001*
	2	68.63 (55, 76.27)	64.63 (33.25, 69.25)	0 (0, 2)	<0.001*
	3	68.94 (55.66, 81.70)	55.93 (39.89, 67.93)	0 (0, 5.51)	<0.001*
P-value (Friedman test)		0.122	0.253	N/A	
RCS	1	76.52 (61.31, 83.15)	65.91 (46.14, 87.25)	0 (0, 0)	<0.001*
	2	78 (63.50, 84.51)	70.17 (42, 85)	0 (0, 0)	<0.001*
	3	75.33 (67.04, 83.99)	76.85 (39.46, 87.72)	0 (0, 0)	<0.001*
P-value (Friedman test)		0.053	0.790	N/A	
SubRe	1	97.50 (85, 100)	82.99 (59.86, 92.78)	0 (0, 2.72)	<0.001*
	2	97.50 (88.13, 100)	76.50 (63.50, 95.50)	0 (0, 6.14)	<0.001*
	3	97.50 (92.50, 100)	77 (61.50, 100)	0 (0, 8.64)	<0.001*
P-value (Friedman test)		0.931	0.969	N/A	
ODT	1	57.69 (34.51, 74.96)	48.12 (28.10, 68.14)	32.27 (0, 38.12)	<0.001*
	2	60.40 (46.39, 90.55)	40.50 (20, 61)	36.75 (0, 46)	<0.001*
	3	69.57 (43.14, 78.09)	54.98 (54.98, 54.98)	37.62 (0, 58.97)	<0.001*
P-value (Friedman test)		0.151	0.135	0.433	
ST	1	63.55 (53.10, 78.23)	39.36 (15.46, 59.38)	34.45 (0, 47.98)	<0.001*
	2	65.50 (55.02, 79.37)	46.50 (31.50, 58)	41.23 (7.01, 52.96)	<0.001*
	3	72.97 (58.08, 83.71)	57.10 (20.85, 59.40)	48.93 (5.07, 48,17)	<0.001*
P-value (Friedman test)		0.015^♭^	0.838	0.918	

Among the ADLs, PBS was identified as the most difficult one since it was the only ADL presenting a median of 0 for all light intensities for both VG2 and VG3. Moreover, the PBS task showed the highest percentage of scores equal to 0, taking into account all lighting settings, namely, 85.42% of the VG3 participants and 70.83% of the VG2 participants were unable to complete this ADL (score = 0). On the other hand, ST was identified as the easiest ADL since 27.08% only of the VG3 participants had a score equal to 0, and the median for VG3 ranged between 34.45 and 48.93.

In addition, all ADLs demonstrated high test-retest reliability with all ICCs above 0.80 (Table 5).

**Table 5:** Test-retest ICCs and repeatability limits of agreement (LoAs) of ADL scores for the three validation groups. ICCs: two-way mixed model with measures of consistency, single rating Lighting Setting 1: 25 fc/3000 K; Lighting Setting 2: 50 fc/4000 K; Lighting Setting 3: 75 fc/6000 K ADLs: activities of daily living; BR: Book Reading; CES: Cellular Entry Search; CI: confidence interval; CM: Cellular Message; DR: Drops bottle Reading; fc: foot candles; ICCs: intraclass correlation coefficients; K: kelvins; N/A: not applicable, ODT: Open Door Test; PBS: Phone Book Search; RCS: Reading Computer Screen; ST: Screwdriver Test; SubRe: Subtitles Reading; SupRe: Supermarket Receipt; VG: validation group

ADLs	Lighting settings	VG1	VG2	VG3
		ICC (95% CI)	ICC (95% CI)	ICC (95% CI)
PBS	1	0.840 (0.707, 0.912)	0.809 (0.464, 0.933)	N/A
	2	0.877 (0.800, 0.931)	0.974 (0.926, 0.991)	N/A
	3	0.906 (0.830, 0.947)	0.918 (0.771, 0.971)	0.805 (0.436, 0.932)
SupRe	1	0.809 (0.581, 0.938)	0.804 (0.255, 0.969)	0.749 (0.291, 0.912)
	2	0.875 (0.764, 0.932)	0.925 (0.480, 0.980)	0.918 (0.754, 0.972)
	3	0.813 (0.664, 0.896)	0.841 (0.297, 0.952)	0.942 (0.798, 0.981)
BR	1	0.901 (0.796, 0.949)	0.872 (0.130, 0.938)	N/A
	2	0.987 (0.965, 0.994)	0.950 (0.238, 0.989)	N/A
	3	0.953 (0.917, 0.974)	0.922 (0.440, 0.979)	0.881 (0.633, 0.959)
DR	1	0.810 (0.640, 0.897)	0.810 (0.640, 0.897)	N/A
	2	0.875 (0.771, 0.931)	0.903 (0.652, 0.968)	N/A
	3	0.882 (0.790, 0.934)	0.863 (0.480, 0.956)	0.866 (0.411, 0.980)
CM	1	0.862 (0.672, 0.959)	0.833 (0.416, 0.908)	0.815 (0.405, 0.938)
	2	0.835 (0.698, 0.910)	0.964 (0.845, 0.989)	0.944 (0.816, 0.981)
	3	0.829 (0.686, 0.907)	0.867 (0.581, 0.955)	0.954 (0.806, 0.986)
CES	1	0.874 (0.759, 0.933)	0.821 (0.481, 0.938)	0.884 (0.668, 0.959)
	2	0.926 (0.864, 0.959)	0.964 (0.894, 0.988)	0.969 (0.911, 0.989)
	3	0.913 (0.841, 0.953)	0.969 (0.909, 0.989)	0.971 (0.913, 0.990)
RCS	1	0.869 (0.764, 0.927)	0.886 (0.676, 0.960)	N/A
	2	0.891 (0.806, 0.939)	0.964 (0.899, 0.987)	N/A
	3	0.915 (0.847, 0.952)	0.979 (0.940, 0.993)	0.879 (0.635, 0.961)
SubRe	1	0.972 (0.940, 0.986)	0.951 (0.860, 0.983)	0.932 (0.800, 0.977)
	2	0.991 (0.982, 0.995)	0.986 (0.961, 0.995)	0.937 (0.823, 0.978)
	3	0.990 (0.980, 0.994)	0.989 (0.968, 0.996)	0.997 (0.992, 0.999)
ODT	1	0.884 (0.658, 0.957)	0.886 (0.650, 0.961)	0.888 (0.644, 0.966)
	2	0.924 (0.812, 0.970)	0.929 (0.807, 0.977)	0.937 (0.801, 0.981)
	3	0.886 (0.717, 0.955)	0.901 (0.853, 0.976)	0.983 (0.946, 0.995)
ST	1	0.816 (0.583, 0.943)	0.829 (0.428, 0.935)	0.932 (0.798, 0.977)
	2	0.852 (0.735, 0.918)	0.913 (0.758, 0.969)	0.831 (0.490, 0.944)
	3	0.823 (0.656, 0.905)	0.852 (0.579, 0.948)	0.921 (0.762, 0.974)

Correlation analysis was performed between each ADL score and binocular UNVA (Table 6). All ADL scores demonstrated a high correlation with UNVA in all lighting settings. Specifically, SupRe, BR, DR, and CM presented excellent negative correlation with UNVA (r < -0.8 in the three lighting settings, p < 0.001); PBS, CES, RCS, and SubRe demonstrated r between -0.8 and -0.6 (p < 0.001); and ODT and ST had a sufficient negative correlation with UNVA (r > - 0.6, p < 0.01 in ODT, p < 0.001 in ST).

**Table 6:** Correlation between each ADL score and binocular UNVA. Pearson correlation ADLs: activities of daily living; BR: Book Reading; CES: Cellular Entry Search; CM: Cellular Message; DR: Drops bottle Reading; fc: foot candles; K: kelvins; ODT: Open Door Test; PBS: Phone Book Search; RCS: Reading Computer Screen; ST: Screwdriver Test; SubRe: Subtitles Reading; SupRe: Supermarket Receipt

ADLs	Lighting settings					
	25 fc/3000 K		50 fc/4000 K		75 fc/6000 K	
	r	P-value	r	P-value	r	P-value
PBS	-0.67	<0.001	-0.71	<0.001	-0.75	<0.001
SupRe	-0.80	<0.001	-0.81	<0.001	-0.79	<0.001
BR	-0.84	<0.001	-0.85	<0.001	-0.84	<0.001
DR	-0.84	<0.001	-0.85	<0.001	-0.84	<0.001
CM	-0.82	<0.001	-0.84	<0.001	-0.84	<0.001
CES	-0.75	<0.001	-0.77	<0.001	-0.75	<0.001
RCS	-0.76	<0.001	-0.76	<0.001	-0.74	<0.001
SubRe	-0.75	<0.001	-0.73	<0.001	-0.74	<0.001
ODT	-0.50	0.002	-0.51	0.002	-0.50	<0.001
ST	-0.57	<0.001	-0.56	<0.001	-0.62	<0.001

## Discussion

It is a truism that presbyopia exerts a negative impact on the quality of life and productivity in the majority of middle-aged people. It is no surprise that conservative estimates suggest that the average American citizen is willing to pay a 5 USD premium for every spectacle-free day for his/her near vision activities [7,19]. The intensive research in presbyopia has produced novel therapeutic options; among them are laser-assisted corrections [20-22], corneal inlays [23,24], and premium intraocular lens implantations [25-27]. However, despite the impressive progress, presbyopia is yet to be fully addressed.

Presbyopia is a multifactorial pathological entity that heavily depends on environmental lighting conditions. Recently published reports suggested an almost linear correlation between visual performance when reading a book and light intensity in patients who underwent pseudophakic presbyopic correction [12]. Lighting societies attempt to address the lighting needs of the human eye with the introduction of lighting directives both for indoor and outdoor settings. However, these directives are based primarily on subjective reports from the research subjects and phasmatoscopic examinations in enucleated eyes, not on actual experimental data about the visual performance in the proposed settings [15,28]. In fact, there is growing evidence that certain prevalent lighting directives result in suboptimal visual capacity and others in energy pollution and wasting [15,29].

It becomes apparent that to identify the actual lighting needs of the presbyopic patient, the following methodological framework would be necessary: a) a validated experimental facility that would secure user-defined environmental illuminance conditions and b) an assessment of the presbyopic patient’s visual performance in a series of ADLs that simulate common daily tasks in the different user-defined illuminance conditions [12,15,17,30]. However, prior to any comparative study, the overall experimental framework has to be validated. Specifically, since it is known that presbyopia gradually reduces near vision capacity and the overall performance of the patient in near and intermediate vision activities and that light intensity correlates positively with visual performance, the proposed experimental framework should differentiate study participants both in terms of near visual acuity and illuminance.

Indeed, that was the objective of the present validation study. A hospital ward was transformed into a high-end artificial lighting facility that provides uniform illuminance conditions both in terms of light intensity and light temperature. We selected three prevalent lighting combinations that are commonly encountered in home and work settings (25 fc/3000 K, 50 fc/4000 K, and 75 fc/6000 K), and three groups of study participants were populated according to their presbyopia (VG1: no presbyopia/0.0-0.1 logMAR; VG2: mild presbyopia/0.4 logMAR; and VG3: advanced presbyopia/0.7 logMAR). All participants had to address a series of ADLs that simulate common daily tasks in all three lighting conditions. Performance scoring in each ADL was done using two nonlinear mathematical models as described in the methods section that evaluate time duration and potential errors.

All proposed ADLs perfectly differentiated study groups, suggesting that they accurately simulate near vision-demanding tasks. As expected, VG1 demonstrated the best median ADL scores in all ADLs and lighting settings, followed by VG2. VG3 presented the worst scores in all ADLs and lighting settings, suggesting increased difficulty in addressing the corresponding tasks.

Among the ADLs, PBS was identified as the most difficult, presenting a median of 0 for all light intensities for both VG2 and VG3, while ST was identified as the easiest ADL with a median for VG3 between 34.45 and 48.93.

As regards the ADL performance of each validation group in the three different lighting settings, it was observed that ADLs using printed material, such as PBS, SupRe, BR, and DR, were clearly influenced by the lighting conditions since significant differences were observed among the three lighting settings for all study groups. Additionally, these tasks showed a monotonical increase in ADL scores as the light intensity and temperature increased in all validation groups; the lighting settings of 25 fc/3000 K demonstrated the lowest ADL scores, while 75 fc/6000 K demonstrated the highest scores. Thus, 25 fc/3000 K seemed to be the most difficult and demanding lighting condition, and 75 fc/6000 K was the easiest and most comfortable one. On the other hand, ADLs using digital screens, such as CM, CES, RCS, and SubRe, and ADLs needing manual dexterity, such as ODT and ST, were not clearly influenced by the lighting conditions, since no significant differences were observed among the three lighting settings in each validation group. In addition, these tasks showed no monotonical increase in ADL scores as the light intensity and temperature increased in all validation groups. A possible explanation is that digital screen-related tasks (i.e., using a smartphone or watching television) are influenced primarily by the brightness and resolution of the screen and not by the environmental illuminance.

Further to adequate validity in differentiating the performance of presbyopic patients in ADLs, our proposed facility demonstrated sufficient test-retest reliability. All test-retest measurements presented high ICCs (above 0.80) for the 15-day time window, which ensured that no significant changes in the vision-related and/or systemic functional status of the participants would take place.

## Conclusions

To our knowledge, this is the first study that objectively compares normophakic participants with different levels of near vision capacity in different illuminance scenarios measuring their performance in a series of ADLs.

Both reliability and validity tests suggested that our methodology is valid for group comparisons. Visual performance in ADLs that used printed material was clearly influenced by the lighting conditions, in contrast to ADLs that used digital screens or required manual dexterity. The lighting setting of 25 fc/3000 K was the most demanding lighting condition, while the lighting setting of 75 fc/6000 K was the most comfortable one. Reading in a phone book was found to be the most demanding near vision daily activity, while screwing with a screwdriver was the easiest ADL.

Our proposed experimental facility could be used not only in naive presbyopic subjects but also in patients who underwent surgical correction of presbyopia, providing an objective assessment of visual performance and prospectively of the efficacy of the surgical intervention. Moreover, in larger cohorts of patients that allow regression analyses, optimal illuminance conditions may be identified that maximize the visual performance of the presbyopic patients and at the same time do not contribute to energy-wasting or energy pollution.

## References

[1] Balgos MJ, Vargas V, Alió JL (2018). Correction of presbyopia: an integrated update for the practical surgeon. Taiwan J Ophthalmol.

[2] Charman WN (2008). The eye in focus: accommodation and presbyopia. Clin Exp Optom.

[3] Atchison DA (1995). Accommodation and presbyopia. Ophthalmic Physiol Opt.

[4] Patel I Research Fellow, West SK El-Maghraby Professor of Preventive Ophthalmology (2007). Presbyopia: prevalence, impact, and interventions. Community Eye Health.

[5] Ramke J, Palagyi A, Naduvilath T, du Toit R, Brian G (2007). Prevalence and causes of blindness and low vision in Timor-Leste. Br J Ophthalmol.

[6] Frick KD, Joy SM, Wilson DA, Naidoo KS, Holden BA (2015). The global burden of potential productivity loss from uncorrected presbyopia. Ophthalmology.

[7] Luo BP, Brown GC, Luo SC, Brown MM (2008). The quality of life associated with presbyopia. Am J Ophthalmol.

[8] Hutchins B, Huntjens B (2021). Patients' attitudes and beliefs to presbyopia and its correction. J Optom.

[9] Charman WN (2014). Developments in the correction of presbyopia I: spectacle and contact lenses. Ophthalmic Physiol Opt.

[10] Labiris G, Giarmoukakis A, Patsiamanidi M, Papadopoulos Z, Kozobolis VP (2015). Mini-monovision versus multifocal intraocular lens implantation. J Cataract Refract Surg.

[11] Labiris G, Ntonti P, Panagiotopoulou EK (2018). Impact of light conditions on reading ability following multifocal pseudophakic corrections. Clin Ophthalmol.

[12] Xu R, Gil D, Dibas M, Hare W, Bradley A (2016). The effect of light level and small pupils on presbyopic reading performance. Invest Ophthalmol Vis Sci.

[13] Illuminating Engineering Society (2011). The IES lighting handbook, 10th ed.

[14] Labiris G, Panagiotopoulou EK, Taliantzis S, Perente A, Delibasis K, Doulos LT (2021). Lighting standards revisited: introduction of a mathematical model for the assessment of the impact of illuminance on visual acuity. Clin Ophthalmol.

[15] (2022). ReluxInformatik AG Münchenstein. https://reluxnet.relux.com/en/downloads.html.

[16] Labiris G, Ntonti P, Patsiamanidi M, Sideroudi H, Georgantzoglou K, Kozobolis VP (2017). Evaluation of activities of daily living following pseudophakic presbyopic correction. Eye Vis (Lond).

[17] Labiris G, Panagiotopoulou EK, Chatzimichael E, Tzinava M, Mataftsi A, Delibasis K (2020). Introduction of a digital near-vision reading test for normal and low vision adults: development and validation. Eye Vis (Lond).

[18] Labiris G, Panagiotopoulou EK, Duzha E, Tzinava M, Perente A, Konstantinidis A, Delibasis K (2021). Development and validation of a web-based reading test for normal and low vision patients. Clin Ophthalmol.

[19] Maxwell WA, Waycaster CR, D'Souza AO, Meissner BL, Hileman K (2008). A United States cost-benefit comparison of an apodized, diffractive, presbyopia-correcting, multifocal intraocular lens and a conventional monofocal lens. J Cataract Refract Surg.

[20] Reilly CD, Lee WB, Alvarenga L, Caspar J, Garcia-Ferrer F, Mannis MJ (2006). Surgical monovision and monovision reversal in LASIK. Cornea.

[21] Miranda D, Krueger RR (2004). Monovision laser in situ keratomileusis for pre-presbyopic and presbyopic patients. J Refract Surg.

[22] Pallikaris IG, Panagopoulou SI (2015). PresbyLASIK approach for the correction of presbyopia. Curr Opin Ophthalmol.

[23] Limnopoulou AN, Bouzoukis DI, Kymionis GD (2013). Visual outcomes and safety of a refractive corneal inlay for presbyopia using femtosecond laser. J Refract Surg.

[24] Menassa N, Fitting A, Auffarth GU, Holzer MP (2012). Visual outcomes and corneal changes after intrastromal femtosecond laser correction of presbyopia. J Cataract Refract Surg.

[25] Labiris G, Patsiamanidi M, Giarmoukakis A, Kozobolis VP (2015). Patient satisfaction and spectacle independence with the iSert multifocal lens. Eur J Ophthalmol.

[26] Lapid-Gortzak R, Labuz G, van der Meulen IJ, van der Linden JW, Mourits MP, van den Berg TJ (2015). Straylight measurements in two different apodized diffractive multifocal intraocular lenses. J Refract Surg.

[27] Kim JS, Jung JW, Lee JM, Seo KY, Kim EK, Kim TI (2015). Clinical outcomes following implantation of diffractive multifocal intraocular lenses with varying add powers. Am J Ophthalmol.

[28] Boettner EA, Wolter JR (1962). Transmission of the ocular media. Investig Ophthalmol Vis Sci.

[29] Papalambrou A, Doulos LT (2019). Identifying, examining, and planning areas protected from light pollution. The case study of planning the first national dark sky park in Greece. Sustainability.

[30] Owsley C, Sloane M, McGwin G Jr, Ball K (2002). Timed instrumental activities of daily living tasks: relationship to cognitive function and everyday performance assessments in older adults. Gerontology.

